# Supplementary material for: Habitat-specific trends in taxonomic, functional, and phylogenetic diversity in European plant communities over a century
Source: Nat Commun. 2026 May 8;17:4208. doi: 10.1038/s41467-026-72112-5 (PMC13156317; doi:10.1038/s41467-026-72112-5)
Supplement: Supplementary file 1 — Supplementary Information [file 41467_2026_72112_MOESM1_ESM.pdf]

Supplementary Information

Habitat-specific trends in taxonomic, functional, and phylogenetic diversity in European plant communities over a century

Stephan Kambach*, Ute Jandt, Alicia Teresa Rosario Acosta, Jose Manuel Álvarez-Martínez, Irena Axmanová, Manuele Bazzichetto, Erwin Bergmeier, Markus Bernhardt-Römermann, Idoia Biurrun, Gianmaria Bonari, Marta Carboni, Marcos Bergmann Carlucci, Maria Laura Carranza, Bruno Enrico Leone Cerabolini, Alessandro Chiarucci, Milan Chytrý, Gabriella Damasceno, Jürgen Dengler, Michele De Sanctis, Jan Divíšek, Jiří Dolezal, Stefan Dullinger, Franz Essl, Klára Friesová, Veronika Fontana, Emmanuel Garbolino, Michael Glaser, Ana González-Robles, Behlül Güler, Georg J. A. Hähn, Michal Hájek, Tracy Hruska, Estela Illa, Florian Jansen, Steve Jansen, Anke Jentsch, Borja Jiménez-Alfaro, W. Daniel Kissling, Ilona Knollová, Gianalberto Losapio, Udayangani Liu, Jonathan Lenoir, Frederic Lens, Bernd Lenzner, Antonio Perea Martos, Laura Méndez, Julie Messier, Akira S. Mori, Francesca Napoleone, Roger Norum, Alexander Novakovskiy, Renske Onstein, Robin J. Pakeman, Josep Peñuelas, Petr Petřík, Remigiusz Pielech, Bruno X. Pinho, Peter Poschlod, Valerijus Rašomavičius, Christiane Roscher, Christian Rossi, Francesco Maria Sabatini, Brody Sandel, David Schellenberger Costa, Wolfgang Schmidt, Serge Sheremetiev, Tanvir Ahmed Shovon, Marko J. Spasojevic, Nathan G. Swenson, Grzegorz Swacha, Rubén Tarifa, Lubomír Tichý, Marcello Tomaselli, Alicia Valdés, Koenraad Van Meerbeek, Vigdis Vandvik, Kiril Vassilev, Martin Večeřa, Evan Weiher, Thomas Wohlgemuth, Helge Bruelheide

*Corresponding author is Stephan Kambach (stephan.kambach@gmail.com)

Supplementary Box 1. Overview on the relationship between species' niche width estimates and threat status derived from European and national Red Lists.

a) Count table of niche width and threat status classification.

**Threat status**	**Generalist**	**Intermediate**	**Specialist**
Not threatened	107	843	108
Red List	198	1,866	265

b) Relationship between niche width and threat status.

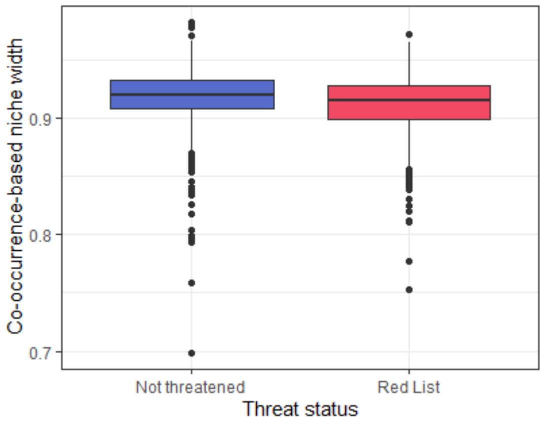

c) Linear model results for the relationship between niche width and threat status.

**Coefficient**	**Estimate**	**Standard error**	**T**	**p**
Intercept	0.92	0.0007	1263.4	< 0.001
Threat status = Red List	-0.004	0.0009	-4.7	< 0.001

Adjusted R^2: 0.006

ANOVA: $F = 21.9$; $df1 = 1$; $df2 = 3,385$; $p < 0.001$

Supplementary Table 1. Traits from the gap-filled dataset from the TRY plant trait database. Those traits that are used for this study are marked in bold.

Abbreviation	Trait description
Chromosome.cDNAcont	Species genotype: chromosome cDNA content_pg
Chromosome.n	Species genotype: chromosome number_dimensionless
Disp.unit.leng	Dispersal unit length_mm
**LDMC**	**Leaf dry mass per leaf fresh mass (leaf dry matter content, LDMC)_g g-1**
Leaf.delta.15N	Leaf nitrogen (N) isotope signature (delta 15N)_per mill
LeafArea.leaf.noPet	Leaf area (in case of compound leaves: leaf, petiole excluded)_mm2
**LeafArea.leaf.undef**	**Leaf area (in case of compound leaves: leaf, undefined if petiole in- or excluded)_mm2**
LeafArea.leaf.wPet	Leaf area (in case of compound leaves: leaf, petiole included)_mm2
LeafArea.leaflet.noPet	Leaf area (in case of compound leaves: leaflet, petiole excluded)_mm2
LeafArea.leaflet.undef	Leaf area (in case of compound leaves: leaflet, undefined if petiole is in- or excluded)_mm2
LeafArea.leaflet.wPet	Leaf area (in case of compound leaves: leaflet, petiole included)_mm2
LeafArea.undef.undef	Leaf area (in case of compound leaves undefined if leaf or leaflet, undefined if petiole is in- or excluded)_mm2
LeafC.perdrymass	Leaf carbon (C) content per leaf dry mass_mg/g
**LeafCN.ratio**	**Leaf carbon/nitrogen (C/N) ratio_g/cm3**
LeafDryMass.single	Leaf dry mass (single leaf)_mg
Leaffreshmass	Leaf fresh mass_g
LeafLength	Leaf length_mm
LeafN	Leaf nitrogen (N) content per leaf dry mass_mg/g
LeafNperArea	Leaf nitrogen (N) content per leaf area_g m-2
**LeafP**	**Leaf phosphorus (P) content per leaf dry mass_mg/g**
**LeafThickness**	**Leaf thickness_mm**
LeafWaterCont	Leaf water content per leaf dry mass (not saturated)_g(W)/g(DM)
LeafWidth	Leaf width_cm
PlantHeight	Plant height (vegetative + generative)
PlantHeight.generative	Plant height generative_m
**PlantHeight.veg**	**Plant height vegetative_m**
**RootingDepth**	**Root rooting depth_m**
Seed.length	Seed length_mm
Seed.num.disp.unit	Seed number per dispersal unit_number
Seed.num.rep.unit	Seed number per reproducton unit_number
SeedGerminationRate	Seed germination rate (germination efficiency)_%
**SeedMass**	**Seed dry mass_mg**
**SLA**	**Leaf area per leaf dry mass (specific leaf area, SLA or 1/LMA)**
SLA.noPet	Leaf area per leaf dry mass (specific leaf area, SLA or 1/LMA): petiole excluded_mm2 mg-1
SLA.photosinthetic.tissues	Leaf area per leaf dry mass (specific leaf area, SLA or 1/LMA) petiole, rhachis and midrib excluded
SLA.undef	Leaf area per leaf dry mass (specific leaf area, SLA or 1/LMA): undefined if petiole is in- or excluded_mm2 mg-1
SLA.wPet	Leaf area per leaf dry mass (specific leaf area, SLA or 1/LMA): petiole included_mm2 mg-1
**SpecificRootLength**	**Root length per root dry mass (specific root length, SRL)_cm/g**
**SpecificRootLength.fine**	**Fine root length per fine root dry mass (specific fine root length, SRL)_cm/g**
**Stem.cond.dens**	**Stem conduit density (vessels and tracheids)_mm-2**
**StemConduitDiameter**	**Stem conduit diameter (vessels, tracheids)_micro m**
StemDens	Stem specific density (SSD) or wood density (stem dry mass per stem fresh volume)_g/cm3
**StemDiam**	**Stem diameter_m**
Wood.vessel.length	Wood vessel element length; stem conduit (vessel and tracheids) element length_micro m
WoodFiberLength	Wood fiber lengths_micro m
WoodRay.millimetre	Wood rays per millimetre_number

Supplementary Table 2. Inferential statistics for estimated average annual percentage changes, relative to baseline conditions (c.f., Fig. 1). Average trends were calculated with weighted linear models to which each time series contribute according to the logarithm of the number of observations. Degrees of freedom is always no. of time series minus one.

Biodiversity index	Weighted average	Standard error	t statistic	p value	Approximated 95% confidence interval	no. of time series
Cover	0.6934	0.0547	12.675	< 0.001	0.5862–0.8001	57,390
Species richness	0.2181	0.0426	5.122	< 0.001	0.1346–0.3015	57,390
Shannon diversity	0.3141	0.0447	7.021	< 0.001	0.2264–0.4017	57,390
Functional richness	0.3142	0.0553	5.684	< 0.001	0.2059–0.4226	30,904
Functional evenness	0.1429	0.0939	1.523	0.128	-0.0411–0.3269	30,904
Functional divergence	0.1381	0.0322	4.285	< 0.001	0.0749–0.2012	30,903
Faith phylogenetic diversity	0.4315	0.0546	7.90	< 0.001	0.3244–0.5385	53,198
Mean pairwise phylogenetic distance	0.5888	0.0719	8.194	< 0.001	0.4480–0.7297	53,198
Mean nearest taxon distance	0.7022	0.0599	11.724	< 0.001	0.5848–0.8196	53,198
No. of Red List species	0.1418	0.0267	5.318	< 0.001	0.0895–0.1941	57,390
Cover of Red List species	0.3898	0.0790	4.938	< 0.001	0.2351–0.5446	57,390
No. of non-native species	0.2443	0.0277	8.825	< 0.001	0.190–0.2985	57,390
Cover of non-native species	1.2335	0.1382	8.926	< 0.001	0.9626–1.5044	57,390
No. of specialists	-0.0018	0.0246	-0.074	0.941	-0.050–0.0464	55,571
No. of generalists	0.4084	0.0491	8.315	< 0.001	0.3122–0.5048	55,571
Cover of specialists	0.5560	0.2902	1.916	0.0554	-0.0127–1.1248	55,571
Cover of generalists	4.0263	0.5861	6.870	< 0.001	2.8776–5.1751	55,571
Community-weighted mean niche width	0.0039	0.0006	7.102	< 0.001	0.0029–0.005	55,571

Supplementary Table 3. Inferential statistics for estimated average annual trends on the original unit of biodiversity indices. Average trends were calculated with weighted linear models to which each time series contribute according to the logarithm of the number of observations. Degrees of freedom is always no. of time series minus one.

Biodiversity index	Weighted average	Standard error	t statistic	p value	Approximated 95% confidence interval	no. of time series
Cover	0.021	0.017	1.21	0.23	-0.013; 0.054	57,390
Species richness	0.00087	0.0044	0.2	0.84	-0.0077; 0.0095	57,390
Shannon diversity	-0.0032	0.00037	-8.53	< 0.001	-0.0039; -0.0024	57,390
Functional richness	-0.0025	0.0085	-0.29	0.77	-0.019; 0.014	30,904
Functional evenness	-0.00095	0.00021	-4.43	< 0.001	-0.0014; -0.00053	30,904
Functional divergence	8.2E-05	0.00019	0.43	0.67	-0.0003; 0.00046	30,903
Faith phylogenetic diversity	-0.056	0.32	-0.18	0.86	-0.68; 0.57	53,198
Mean pairwise phylogenetic distance	-0.026	0.049	-0.53	0.60	-0.12; 0.070	53,198
Mean nearest taxon distance	0.22	0.062	3.54	< 0.001	0.098; 0.34	53,198
No. of Red List species	-0.00072	0.00033	-2.20	0.03	-0.0014; -8E-05	57,390
Cover of Red List species	-0.019	0.0033	-5.7	< 0.001	-0.025; -0.012	57,390
No. of non-native species	0.00058	0.00031	1.87	0.06	-2.6E-05; 0.0018	57,390
Cover of non-native species	0.022	0.0058	3.79	0.0002	0.010; 0.034	57,390
No. of specialists	-0.0010	0.00024	-4.26	< 0.001	-0.0015; -0.00054	55,571
No. of generalists	0.0035	0.0013	2.75	0.006	0.0010; 0.0061	55,571
Cover of specialists	-0.0065	0.0053	-1.23	0.22	-0.017; 0.0038	55,571
Cover of generalists	-0.095	0.016	-5.89	< 0.001	-0.13; -0.063	55,571
Community-weighted mean niche width	3.6E-05	5.1E-06	6.96	< 0.001	2.6E-05; 4.6E-05	55,571

Supplementary Table 4. Inferential statistics for balanced trends in annual percentage changes of local diversity indices (%, relative to the baseline conditions), calculated with even contributions of each EUNIS level 3 habitat type. Degrees of freedom is always no. of time series minus one.

Biodiversity index	Weighted average	Standard error	t statistic	p value	Approximated 95% confidence interval	no. of time series
Cover	1.01	0.07	15.06	< 0.001	0.88; 1.14	57,390
Species richness	1.44	0.07	20.20	< 0.001	1.30; 1.58	57,390
Shannon diversity	0.84	0,05	17.50	< 0.001	0.75; 0.91	57,390
Functional richness	0.80	0.06	13.82	< 0.001	0.69; 0.91	30,904
Functional evenness	0.22	0.10	2.12	0.034	0.02; 0.42	30,904
Functional divergence	0.10	0.051	1.95	0.052	-0.001; 0.2	30,903
Faith phylogenetic diversity	0.89	0.06	15.56	< 0.001	0.78; 1.004	53,198
Mean pairwise phylogenetic distance	0.48	0.14	3.30	0.001	0.194; 0.76	53,198
Mean nearest taxon distance	-0.17	0.08	-2.10	0.035	-0.33; -0.01	53,198
No. of Red List species	-0.05	0.05	-1.02	0.31	-0.15; 0.05	57,390
Cover of Red List species	0.33	0.23	1.39	0.16	-0.13; 0.79	57,390
No. of non-native species	0.39	0.06	6.46	< 0.001	0.27; 0.51	57,390
Cover of non-native species	1.43	0.16	8.95	< 0.001	1.12; 1.75	57,390
No. of specialists	-0.05	0.03	-1.59	0.11	-0.12; 0.0	55,571
No. of generalists	1.86	0.08	22.90	< 0.001	1.70; 2.02	55,571
Cover of specialists	-0.16	0.06	-2.59	0.018	-0.29; -0.04	55,571
Cover of generalists	3.73	0.27	13.60	< 0.001	3.19; 4.27	55,571
Community-weighted mean niche width	0.015	0.001	18.67	< 0.001	0.01; 0.02	55,571

Supplementary Table 5. Inferential statistics for balanced annual trends on the original unit of biodiversity indices, calculated with even contributions of each EUNIS level 3 habitat type. Degrees of freedom is always no. of time series minus one.

Biodiversity index	Weighted average	Standard error	t statistic	p value	Approximated 95% confidence interval	no. of time series
Cover	0.18	0.019	9.51	< 0.001	0.15; 0.022	57,390
Species richness	0.18	0.0094	19.66	< 0.001	0.17; 0.023	57,390
Shannon diversity	0.011	0.00062	16.89	< 0.001	0.009; 0.012	57,390
Functional richness	0.16	0.013	12.48	< 0.001	0.134; 0.183	30,904
Functional evenness	-0.0013	0.00032	-4.04	< 0.001	-0.002; -0.001	30,904
Functional divergence	-0.00057	0.00028	-2.06	0.039	-0.001; 0	30,903
Faith phylogenetic diversity	10.18	0.65	15.62	< 0.001	8.91; 11.461	53,198
Mean pairwise phylogenetic distance	0.15	0.064	2.33	0.019	0.024; 0.273	53,198
Mean nearest taxon distance	-0.86	0.074	-11.66	< 0.001	-1.008; -0.718	53,198
No. of Red List species	-0.0038	0.00067	-5.73	< 0.001	-0.005: -0.003	57,390
Cover of Red List species	-0.091	0.0073	-12.5	< 0.001	-0.11; -0.076	57,390
No. of non-native species	-0.0016	0.0008	-1.86	0.062	-0.003; 0	57,390
Cover of non-native species	0.0095	0.0089	1.07	0.283	-0.008; 0.027	57,390
No. of specialists	-0.0016	0.00037	-4.29	< 0.001	-0.002; -0.001	55,571
No. of generalists	0.046	0.0023	20.2	< 0.001	0.041; 0.05	55,571
Cover of specialists	-0.034	0.0051	-6.65	< 0.001	-0.044; -0.024	55,571
Cover of generalists	0.15	0.015	9.97	< 0.001	0.118; 0.176	55,571
Community-weighted mean niche width	0.00014	7.3E-06	18.51	< 0.001	0.00012; 0.00015	55,571

Supplementary Table 6. Impact of resurvey design on average annual percentage changes in indices of local plant diversity. Coef – coefficient estimates for separately tested predictors of plot size change (in % from the first to the last observation date) and resampling strategy (permanent vs. semi-permanent) in separate weighted linear models for each biodiversity index (with the observed annual percentage change as the dependent variable). Significant coefficient estimates are highlighted in bold, based on the respective SE – standard error, two-sided Students *t*-tests, and $p < 0.05$. Joint model statistic relate to the joint explanatory power of the two predictors, quantified by the joint explained variation ($R^2 * 100 = \%$) and the adjusted p-value. Grand means for average and balanced diversity trends are shown in Extended Data Table 4.

	Plot size change				Resampling design				Joint model		
**Biodiversity index**	**Coef**	**SE**	**t**	**p**	**Coef**	**SE**	**t**	**p**	**Explained variation (%)**	$F_{(df = 2)}$	**P**
Cover	1.66E-06	1,41E-05	0,12	0,91	-0,17107	0,154057	-1,11046	0,266806	0	0,62	0,54
Species richness	4.89E-06	1,09E-05	0,45	0,66	0,301295	0,119902	2,512839	0,011979	0,01	3,42	0,03
Shannon diversity	9,78E-07	1,15E-05	0,09	0,93	-0,38759	0,125946	-3,07739	0,002089	0,01	4,77	0,01
Functional richness	3,62E-06	1,16E-05	0,31	0,76	0,024723	0,167973	0,147186	0,882986	-0,01	0,07	0,93
Functional evenness	-1,3E-05	1,98E-05	-0,66	0,51	0,529273	0,285211	1,855724	0,063502	0,01	1,80	0,17
Functional divergence	-2E-06	6,79E-06	-0,30	0,77	-0,09117	0,097901	-0,93123	0,351742	0	0,53	0,59
Faith phylogenetic diversity	1,61E-06	1,35E-05	0,12	0,91	-0,10702	0,153143	-0,69879	0,484686	0	0,25	0,78
Mean pairwise phylogenetic distance	-4,3E-06	1,78E-05	-0,24	0,81	-0,8585	0,201467	-4,26126	2,04E-05	0,03	9,33	< 0.001
Mean nearest taxon distance	-1,3E-05	1,48E-05	-0,89	0,38	-0,93565	0,167908	-5,57239	2,52E-08	0,06	16,66	< 0.001
No. of Red List species	5,93E-07	6,85E-06	0,09	0,93	-0,2881	0,075074	-3,83753	0,000124	0,02	7,42	0,001
Cover of Red List species	1,11E-06	2,03E-05	0,05	0,96	-0,68344	0,222307	-3,07432	0,002111	0,01	4,76	0,01
No. of non-native species	-1,1E-05	7,1E-06	-1,53	0,13	0,850678	0,077871	10,92422	9,4E-28	0,2	59,74	< 0.001
Cover of non-native species	-3,1E-05	3,55E-05	-0,87	0,38	1,812411	0,389101	4,65794	3,2E-06	0,03	10,92	< 0.001
No. of specialists	9,88E-06	6,31E-06	1,57	0,12	0,070906	0,069876	1,014731	0,310239	0	1,936	0,14
No. of generalists	-3,8E-06	1,26E-05	-0,30	0,76	-0,09172	0,139395	-0,65798	0,510551	0	0,29	0,75
Cover of generalists	-6,3E-06	0,00015	-0,04	0,97	-4,12866	1,662858	-2,48287	0,013036	0,01	3,13	0,04
Cover of specialists	4,22E-06	7,43E-05	0,06	0,95	-0,65426	0,823343	-0,79464	0,426824	0	0,32	0,73
Community-weighted mean niche width	-5,5E-08	1,42E-07	-0,39	0,70	0,000564	0,001574	0,358234	0,72017	0	0,13	0,88

Supplementary Table 7. Hand-corrected occurrences of potential "doppelganger taxa", which we defined as taxa with different names in subsequent vegetation surveys could belong to the same taxon.

Resurvey project	Resurvey site	"Doppelgaenger taxon"	Changed to	# of changed occurrences
Active Conservation of Dry Grasslands in CE Poland	Rm, Rb	*Galium verum*	*Galium album*	18
DISEQALP		*Anthoxanthum alpinum*	*Anthoxanthum* aggr.	387
		Anthoxanthum odoratum	*Anthoxanthum* aggr.	351
		Anthoxanthum odoratum aggr.	*Anthoxanthum* aggr.	20
		Empetrum hermaphroditum	*Empetrum* aggr.	176
		Empetrum nigrum	*Empetrum* aggr.	55
		Empetrum nigrum aggr.	*Empetrum* aggr.	6
		Sesleria varia aggr.	*Sesleria* aggr.	59
		Sesleria albicans	*Sesleria* aggr.	1,022
forestREplot_EU_023a		*Tilia platyphyllos*	*Tilia cordata*	6
GLORIA_NO-DOV		*Empetrum nigrum subsp. hermaphroditum*	*Empetrum nigrum*	53
		Juncus trifidus subsp. trifidus	*Juncus trifidus*	40
jaidhaus_2020_hschwaiger		*Leucanthemum vulgare*	*Leucanthemum vulgare* aggr.	19
		Leucanthemum ircutianum	*Leucanthemum vulgare* aggr.	50
		Leucanthemum vulgare aggr.	*Leucanthemum vulgare* aggr.	64
Milovice_grazing		*Rubus caesius*	*Rubus sp.*	6
PonorMts	Ponor	*Agrostis stolonifera*	*Agrostis capillaris*	8
		Cirsium vulgare	*Cirsium ligulare*	13
		Euphrasia species	*Euphrasia pectinata*	34
Valdemaqueda_Madrid		*Vicia angustifolia*	*Vicia lathyroides*	151
WS11 NWR Rotenberghang	StrictNR	*Dryopteris dilatate*	*Dryopteris carthusiana* aggr.	5
		Dryopteris carthusiana	*Dryopteris carthusiana* aggr.	29
WS26 Hunstollen-Brache		*Dactylis glomerata*	*Dactylis glomerata* aggr.	509
		Dactylis glomerata subsp. aschersoniana	*Dactylis glomerata* aggr.	16

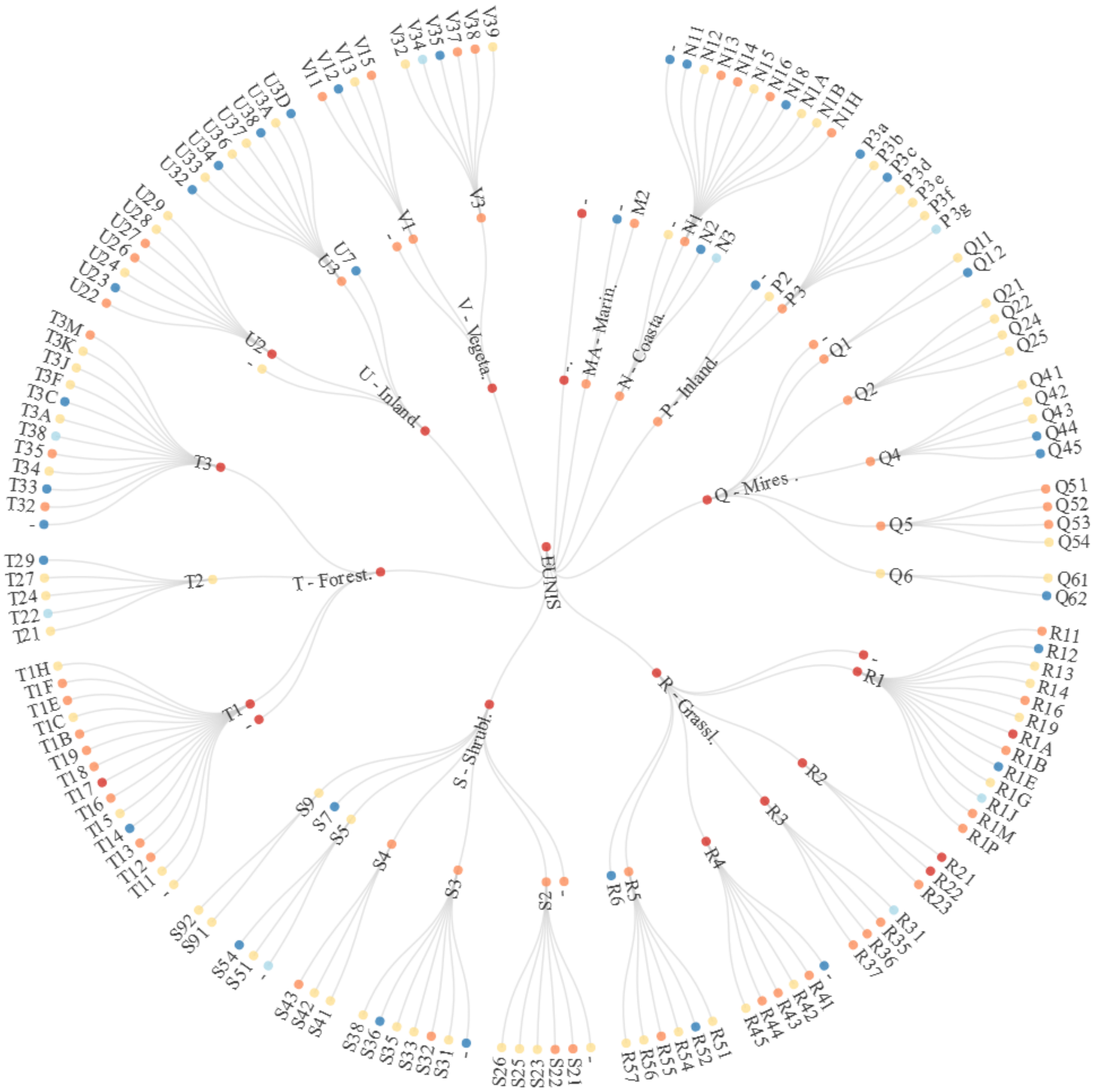

Supplementary Fig. 1. Number of time series within level 1 to 3 of the EUNIS hierarchy of habitat types. Colouring indicates number of time series (assignment on the classification at the first survey date): =1■ <10■ <100■ <1,000■ ≥1,000■.

“-“ shows the number of plots that could not be assigned to a habitat type.

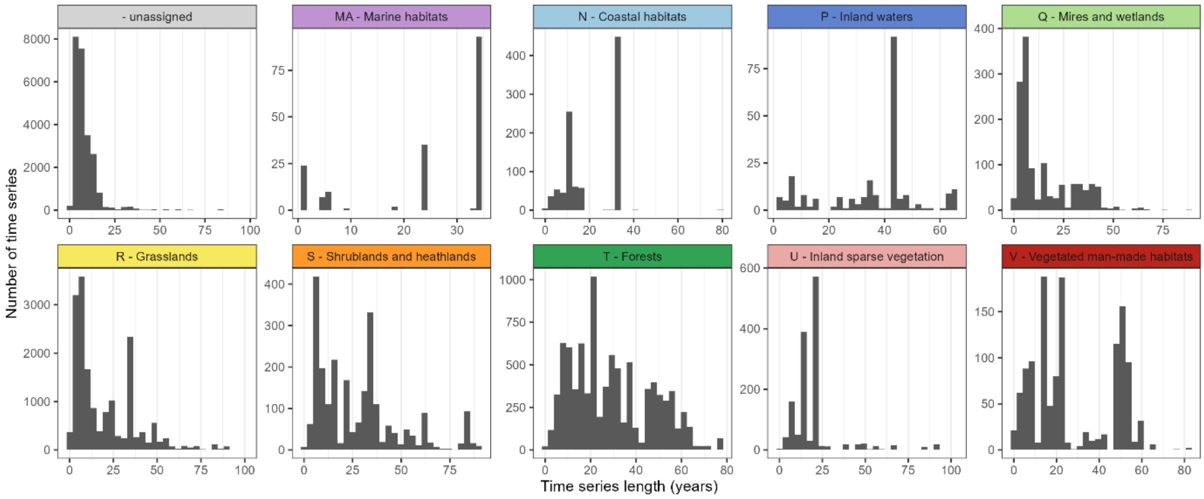

Supplementary Fig. 2. Distribution of time spans per EUNIS level 1 habitat types.

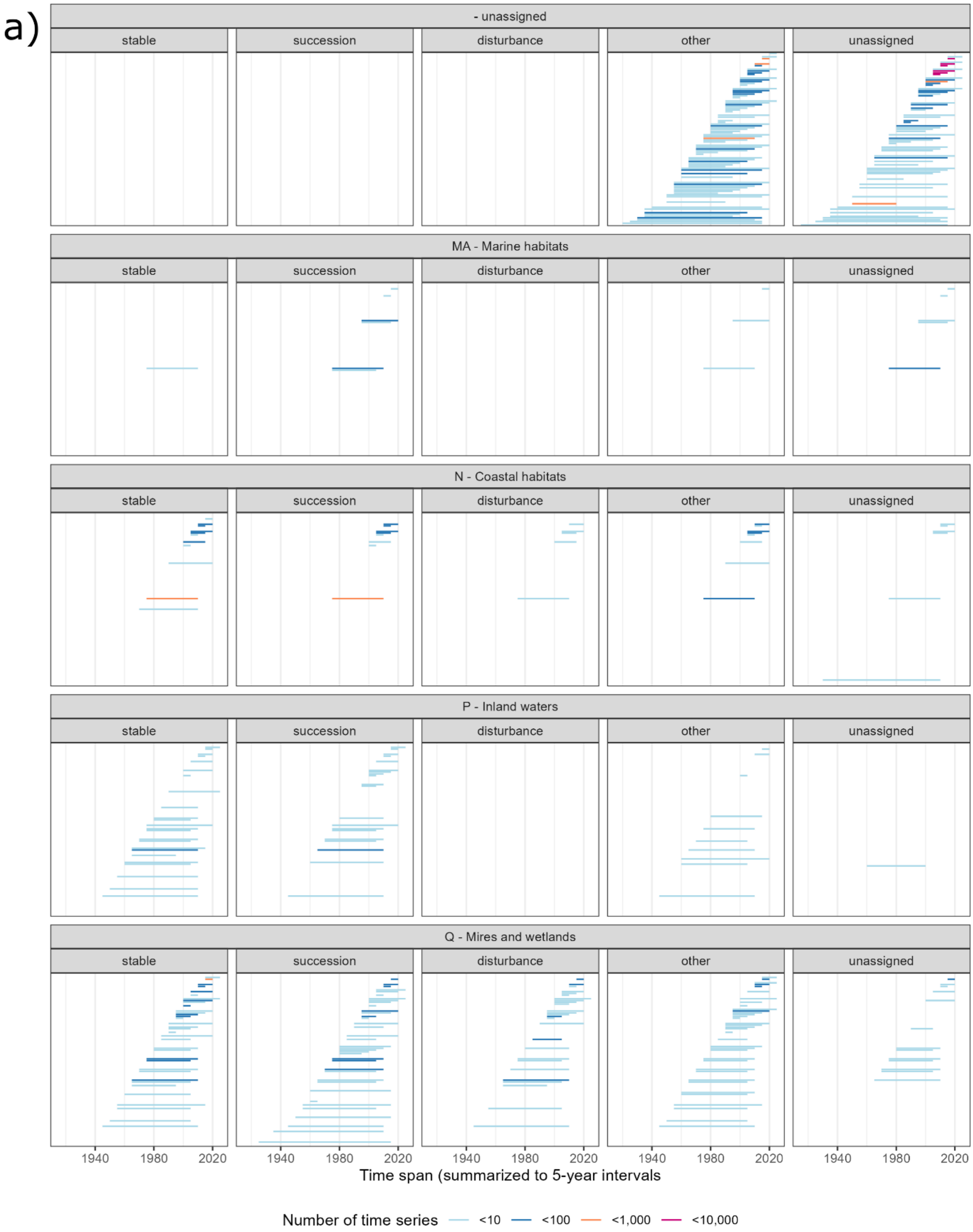
a)
- unassigned
MA - Marine habitats
N - Coastal habitats
P - Inland waters
Q - Mires and wetlands
stable
succession
disturbance
other
unassigned
1940
1980
2020
Time span (summarized to 5-year intervals)
Number of time series
<10
<100
<1,000
<10,000

b)

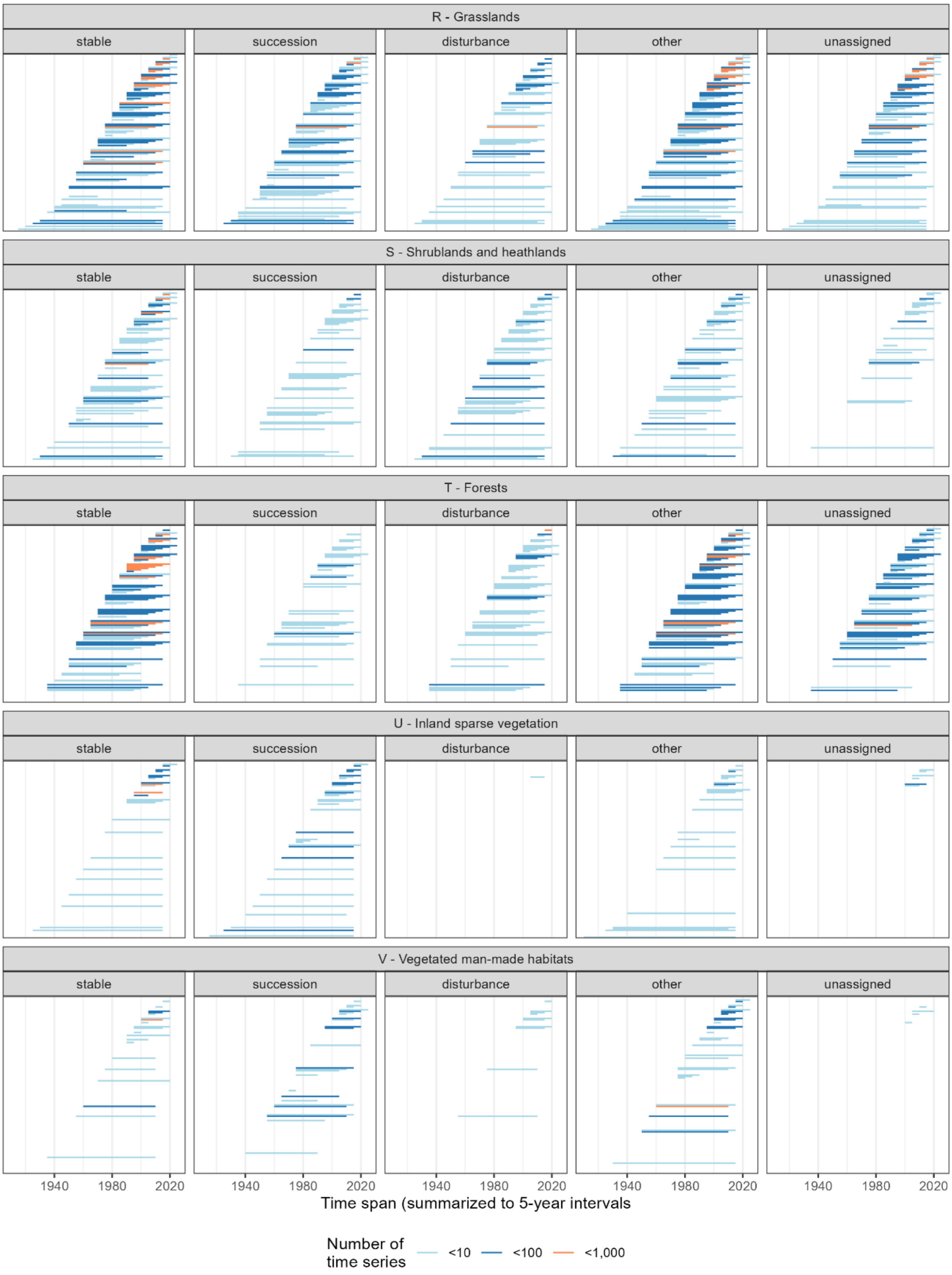

Supplementary Fig. 3. Time span and number of time series per EUNIS level 1 habitat types (aggregated into bins of 5-year intervals).

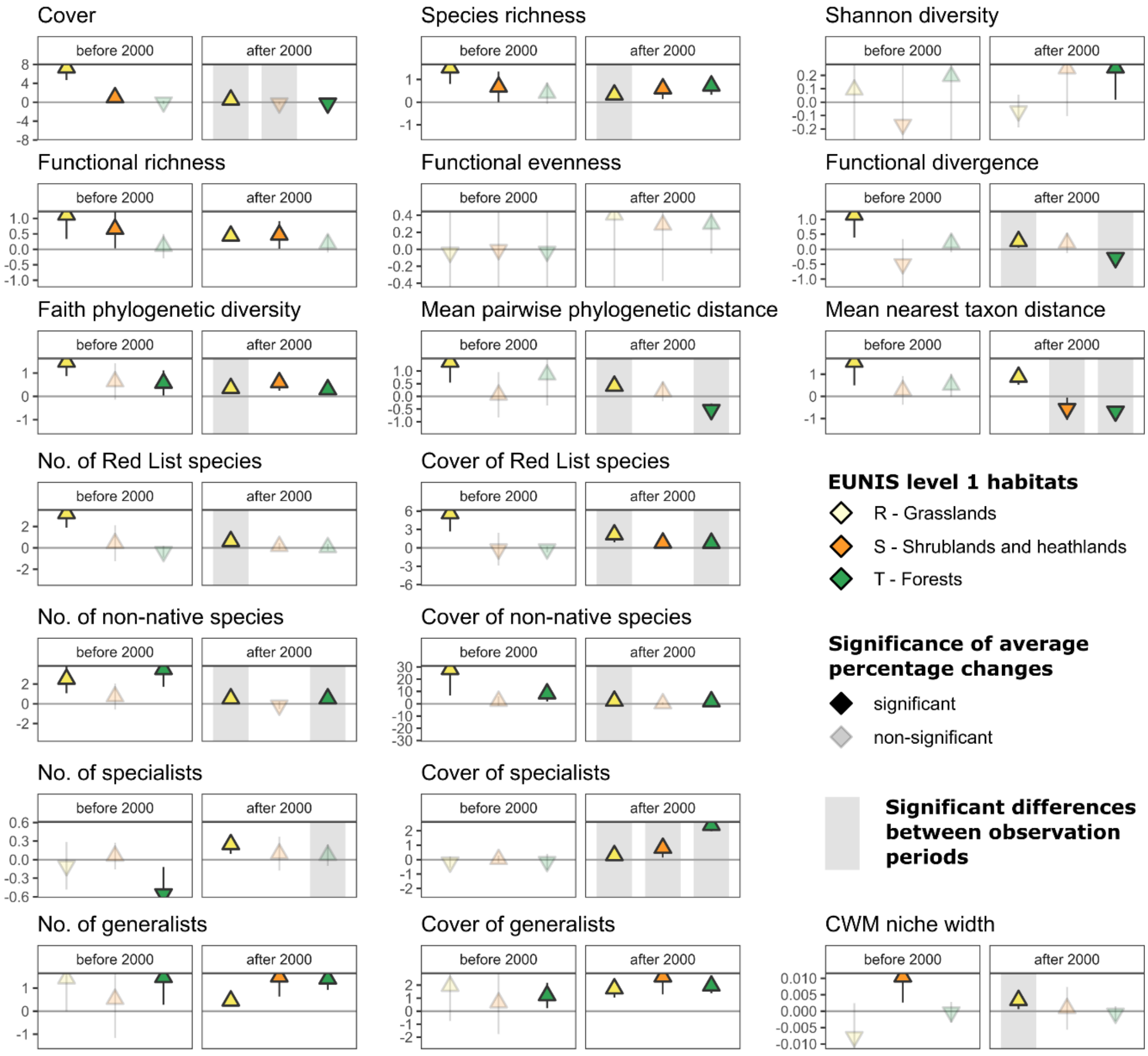

Supplementary Fig. 4. Average annual percentage changes in diversity indices of local plant communities, separated into time series before versus after the year 2000. Subgroup-specific average annual trends were calculated with separate weighted linear models. Significance of the estimated weighted average values was tested with separate two-sided Student's *t*-tests (at $p < 0.05$). Error bars show Wald-approximated 95% confidence intervals, calculated as weighted average ± 1.96 * standard error. Statistics in Supplementary Data 4.

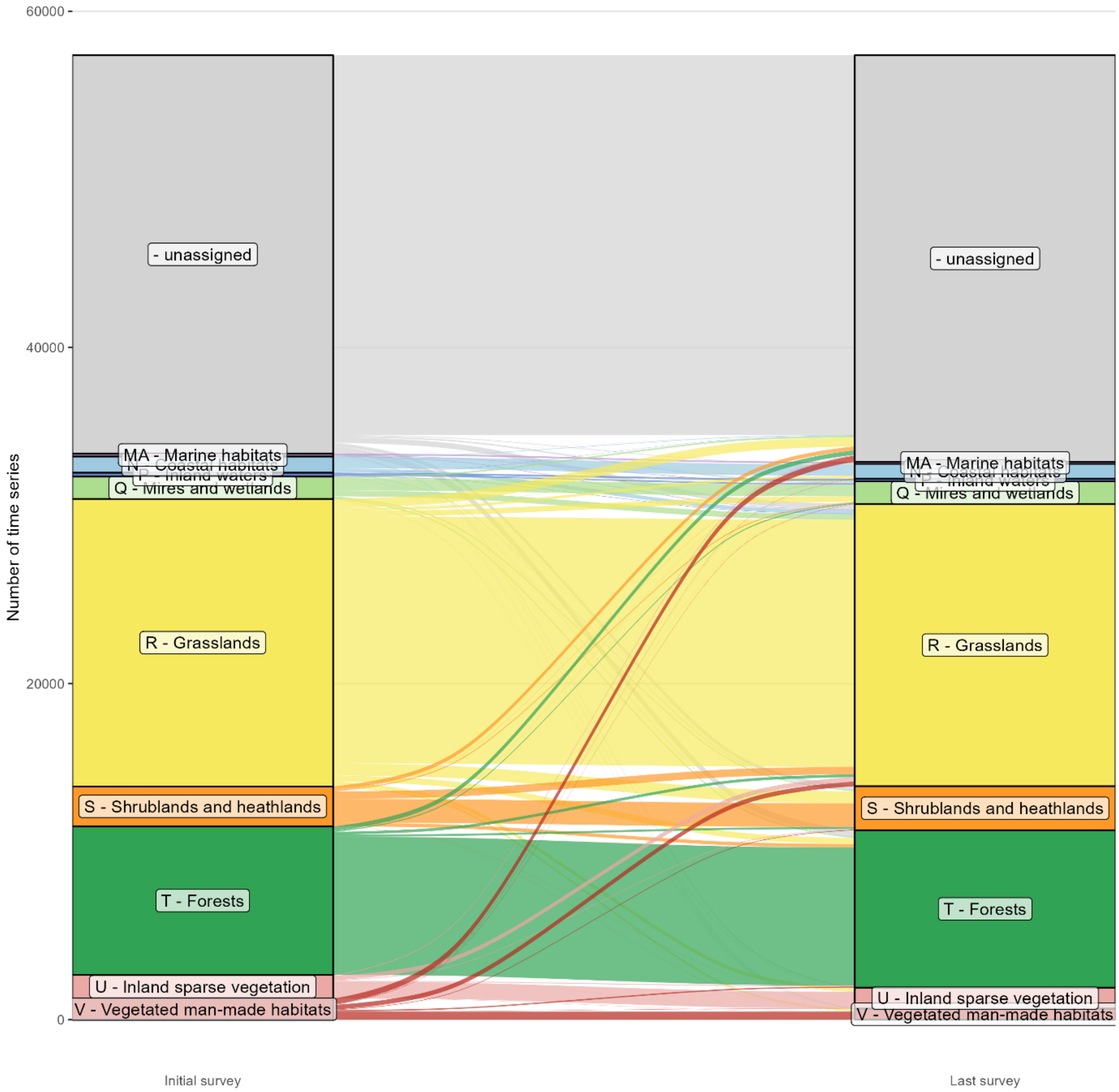

Supplementary Fig. 5. Habitat shifts among EUNIS level 1 habitat types in the ReSurvey Europe time series data, including time series that could not be assigned to a habitat type and time series with stable level 1 habitat types.

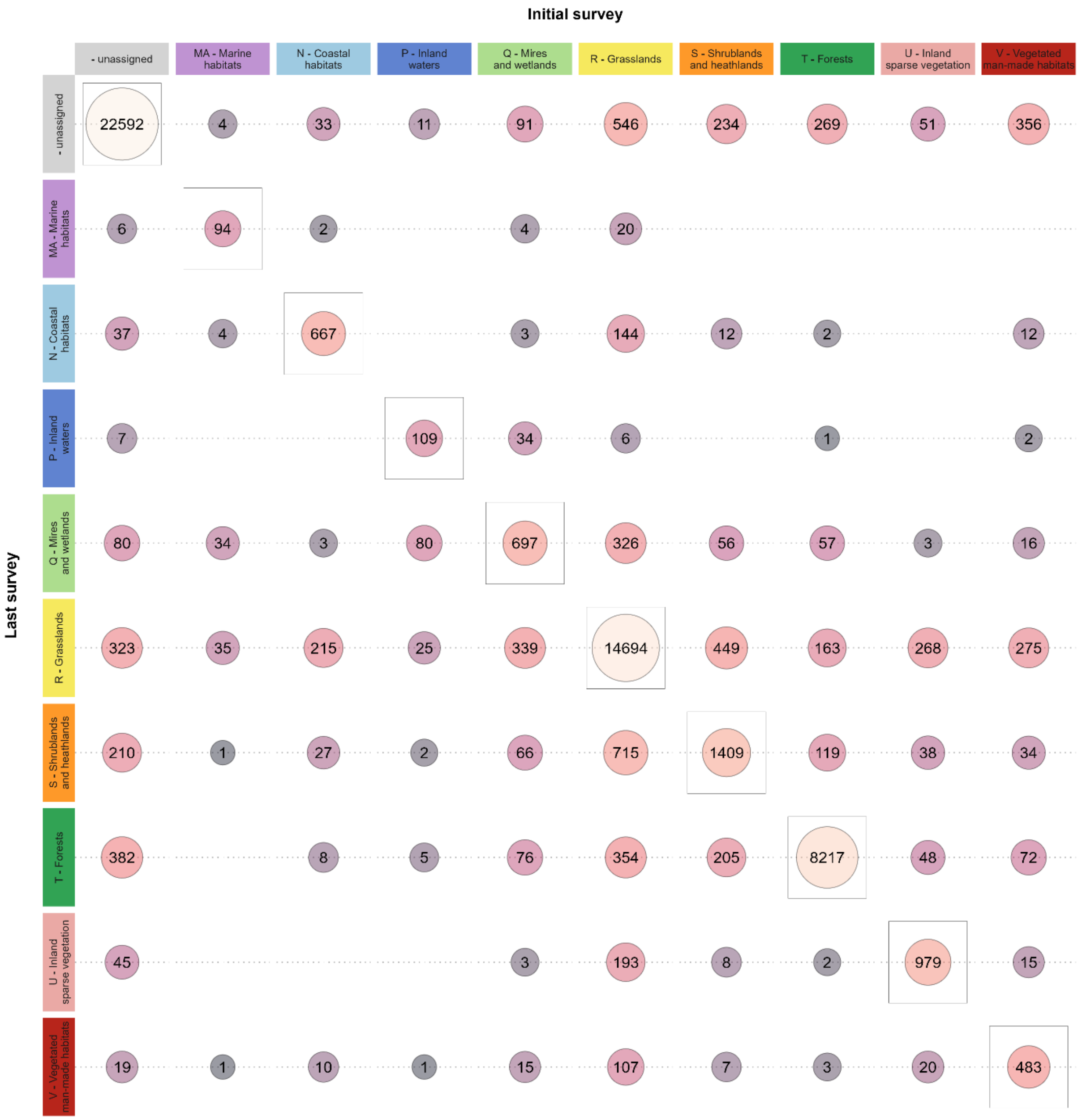

Supplementary Fig. 6. Number and direction of EUNIS level 1 habitat shifts within time series (i.e., differences between vegetation surveys at the first versus the last survey date). Numbers refer to the number of time series (further illustrated by the size and color of circles). Time series in the diagonal (marked with boxes) showed no shift in EUNIS level 1 habitat types.

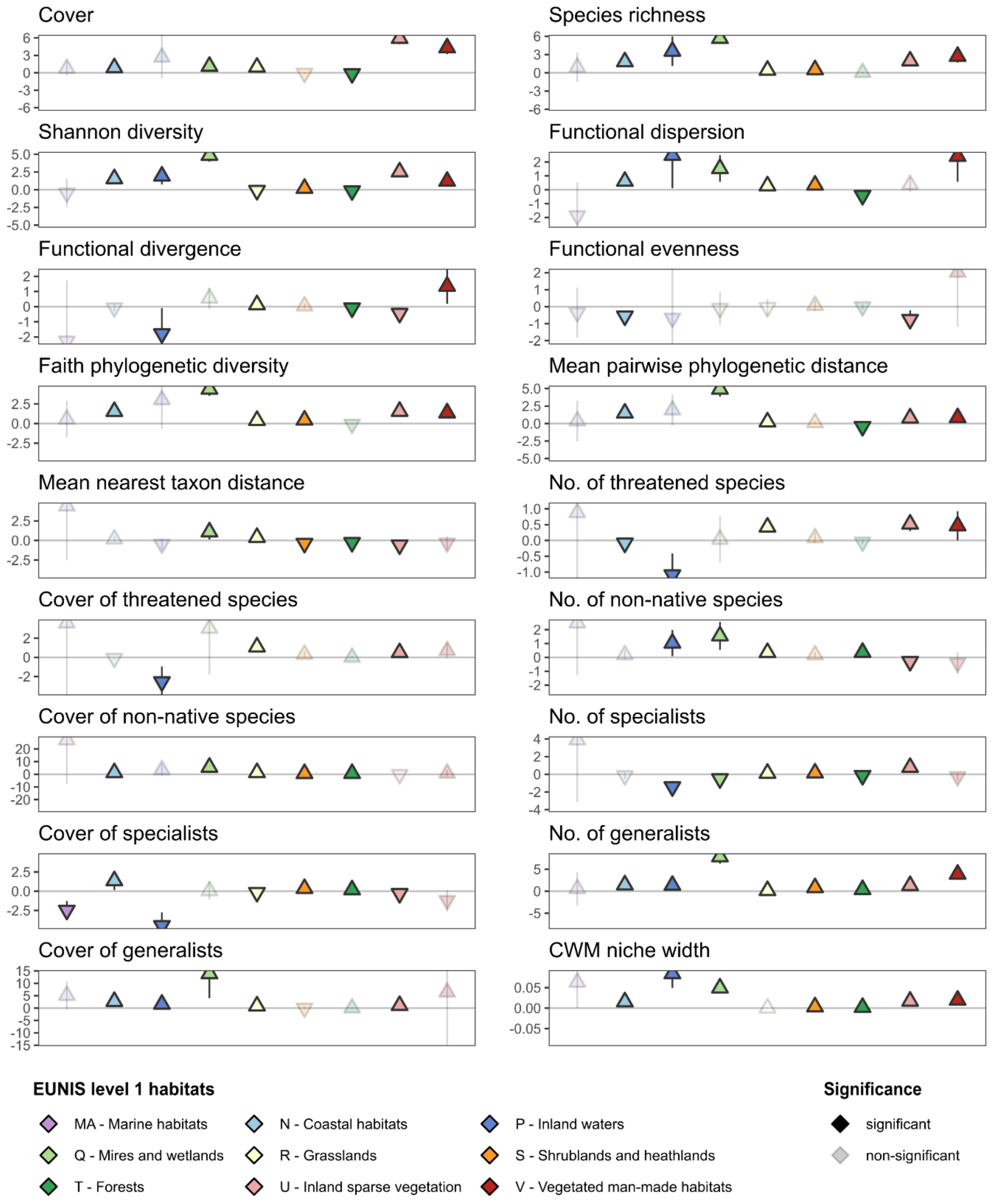

Supplementary Fig. 7. Average annual percentage changes of plant diversity indices within EUNIS level 1 habitat types (estimated with weighted linear models in which each time series contributed according to the logarithm of the number of vegetation surveys). Significance of the estimated weighted average values was tested with separate two-sided Student's *t*-tests (at $p < 0.05$). Error bars show Wald-approximated 95% confidence intervals, calculated as weighted average ± 1.96 * standard error. Statistics in Supplementary Data 6.

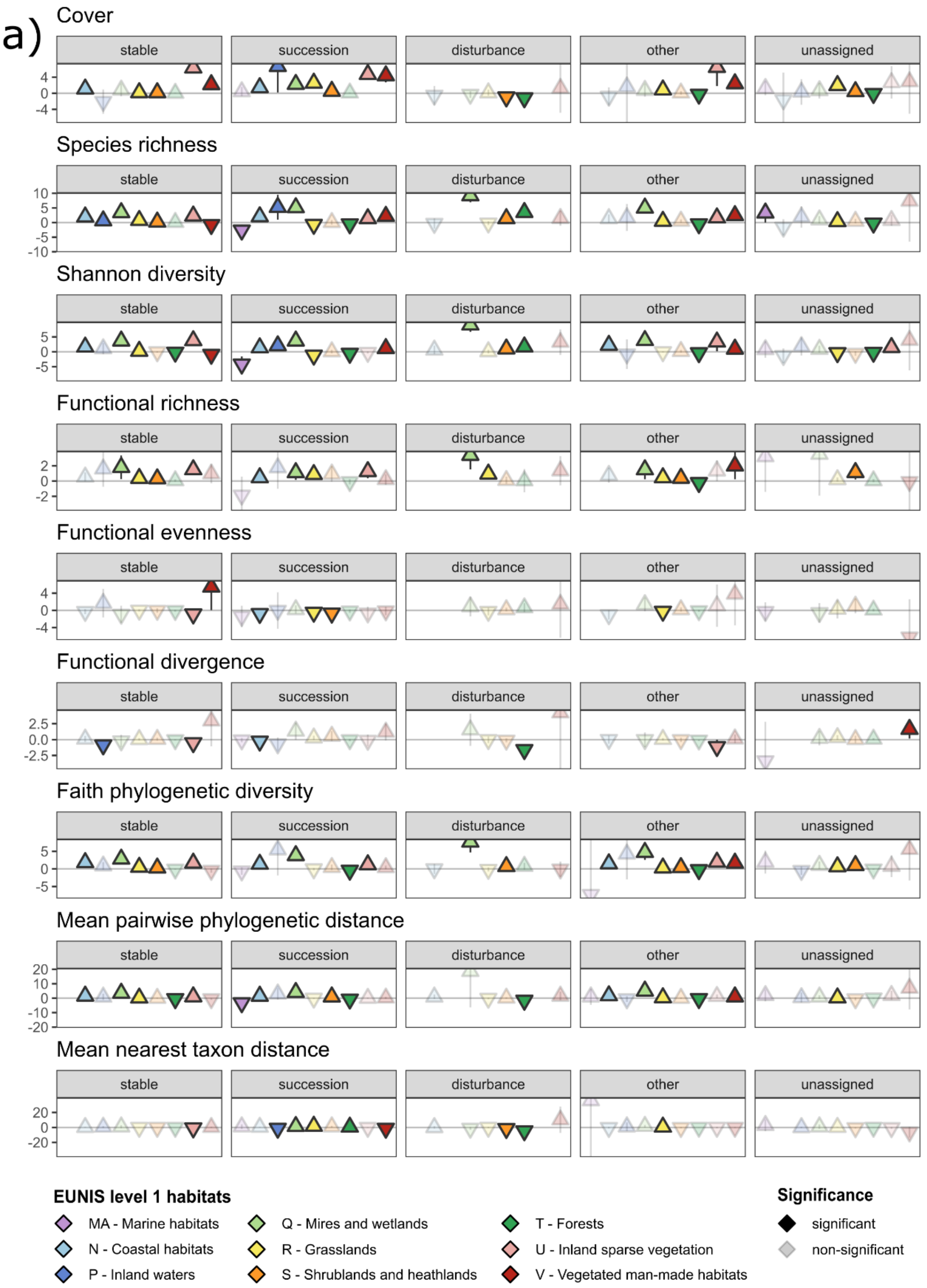

a)
Cover
Species richness
Shannon diversity
Functional richness
Functional evenness
Functional divergence
Faith phylogenetic diversity
Mean pairwise phylogenetic distance
Mean nearest taxon distance
stable
succession
disturbance
other
unassigned
EUNIS level 1 habitats
MA - Marine habitats
N - Coastal habitats
P - Inland waters
Q - Mires and wetlands
R - Grasslands
S - Shrublands and heathlands
T - Forests
U - Inland sparse vegetation
V - Vegetated man-made habitats
Significance
significant
non-significant

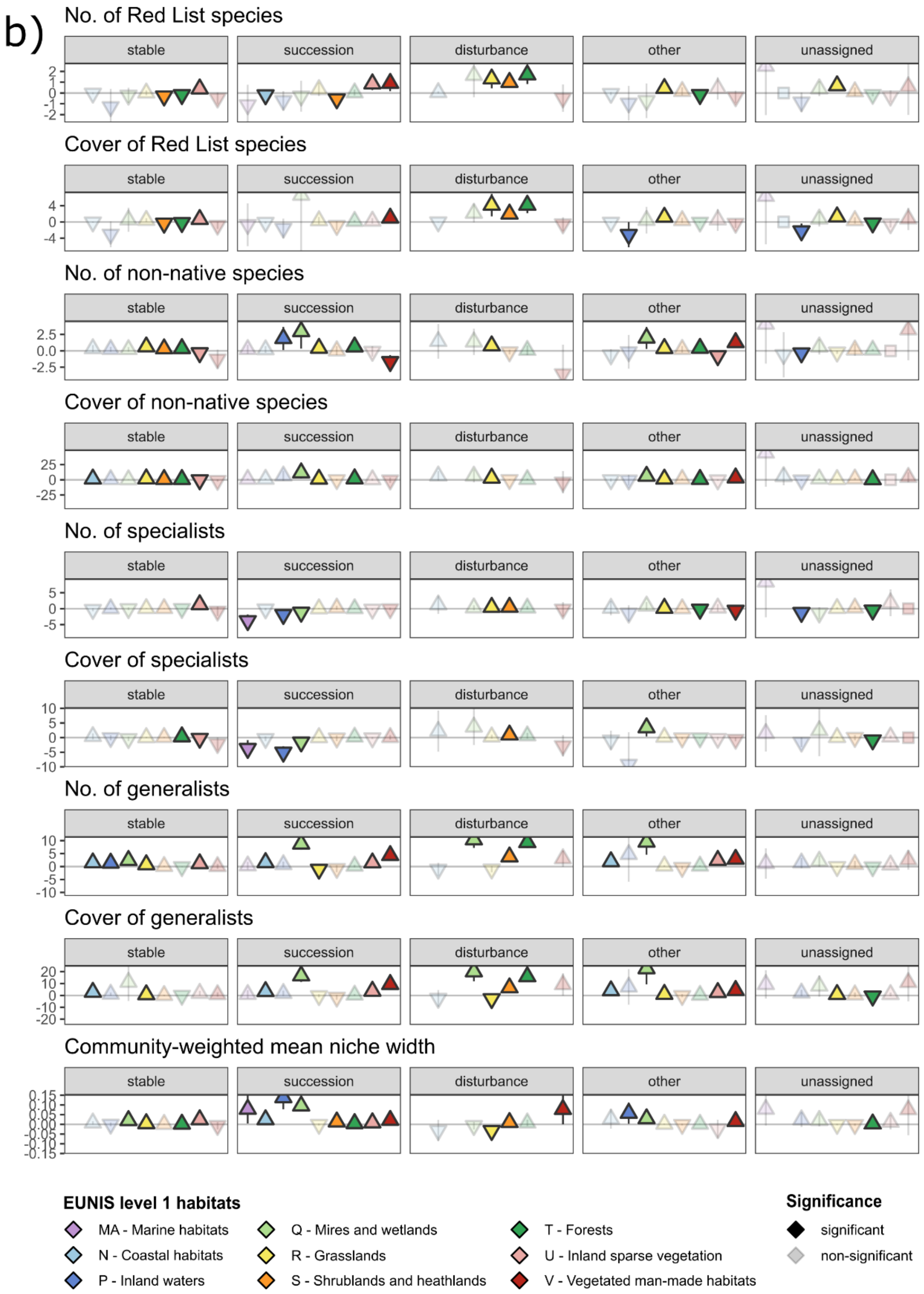

Supplementary Fig. 8. Average annual percentage changes of local plant diversity indices separated by EUNIs level 1 habitat and habitat change trajectory (estimated with weighted linear models in which each time series contributed according to the logarithm of the number of vegetation-plot observations). Habitat-change trajectories were assigned based on shifts in EUNIS level 3 habitat types between the initial versus the last vegetation survey. Significance of the estimated weighted average values was tested with separate two-sided Student's *t*-tests (at $p < 0.05$). Error bars show Wald-approximated 95% confidence intervals, calculated as weighted average ± 1.96 * standard error. Statistics in Supplementary Data 6.

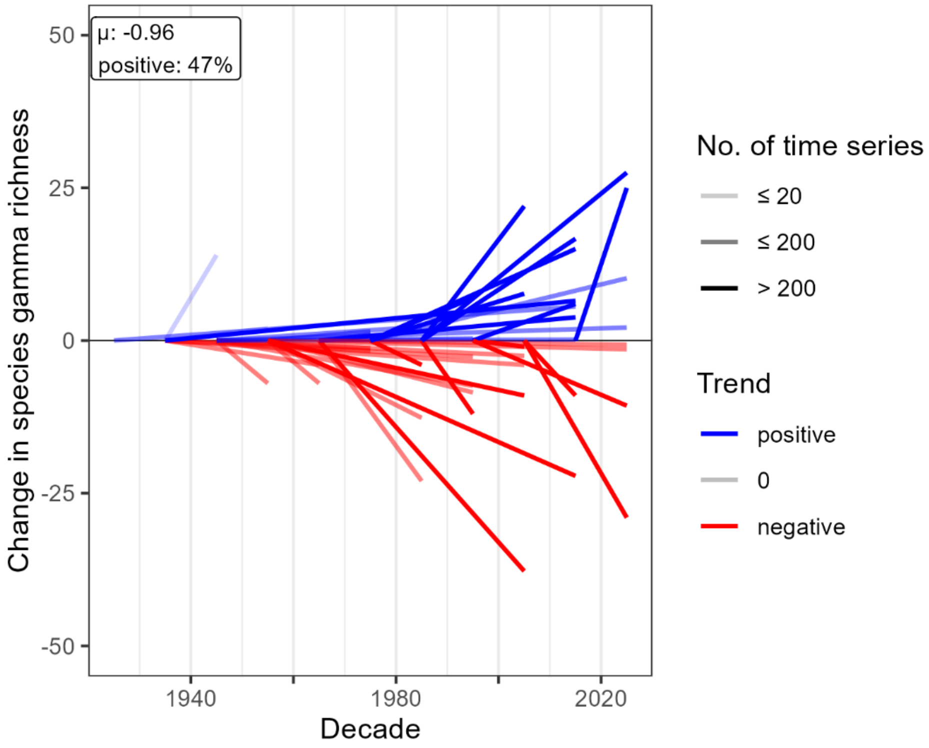

Supplementary Fig. 9. Decadal trends in European gamma diversity across all vegetation-plot time series. To account for temporal trends in sample sizes, each trend line includes only pairs of observations from vegetation plots that were that were surveyed in the focal two decades. In the box, the first number shows the average decadal trend in gamma diversity (with asterisks indicating significance according to a two-sided Students *t*-tests at $p < 0.05$) and the second number shows the proportion of positive versus negative decadal trends (with asterisks indicating significance according to a two-sided binomial test at $p < 0.05$). Statistics in Supplementary Data 7.

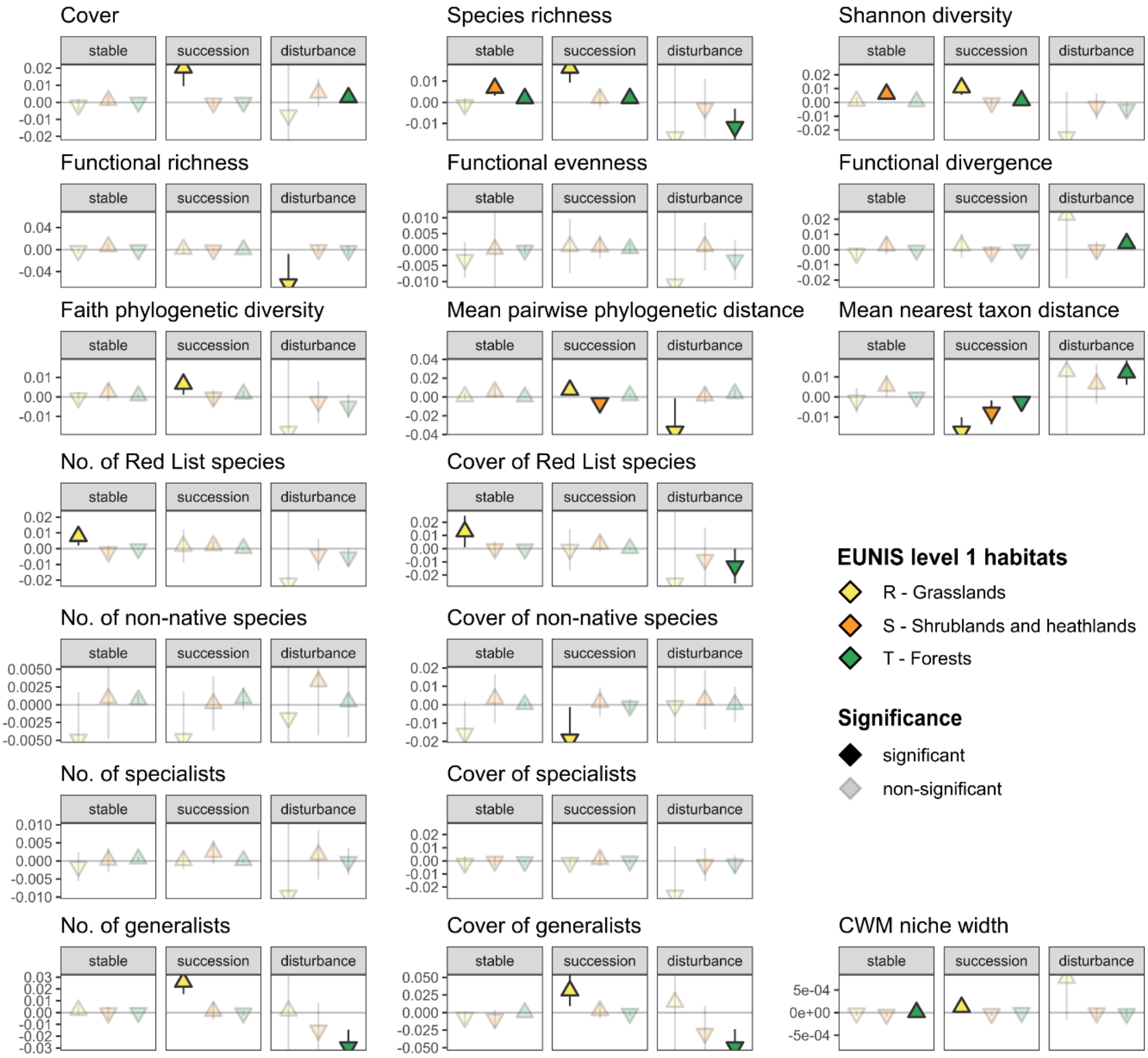

Supplementary Fig. 10. Impact of plot size (in m²) on the obtained average annual percentage changes in diversity indices of local plant communities. Subgroup-specific average annual trends were calculated with weighted linear models. Significance of the estimated weighted average values was tested with separate two-sided Student's *t*-tests (at $p < 0.05$). Error bars show Wald-approximated 95% confidence intervals, calculated as weighted average ± 1.96 * standard error. Statistics in Supplementary Data 8.

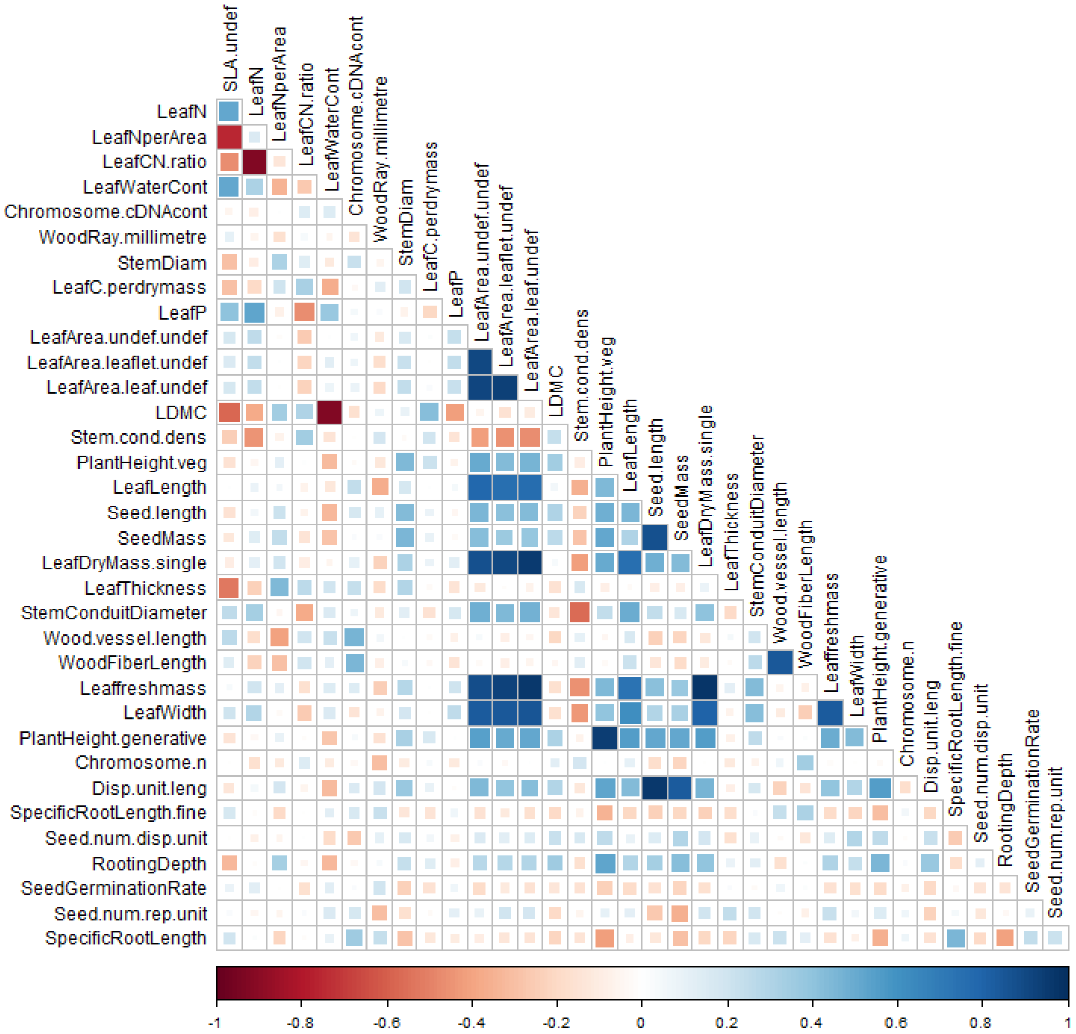

Supplementary Fig. 11. Bivariate correlations among the 46 traits of the gap-filled TRY plant trait database (Kattge et al. 2020), calculated for the subset of 3,834 taxa that occurred in our vegetation-plot time series data. Trait names and descriptions are noted in Table SX3. Correlations are quantified as Pearson's *r*.

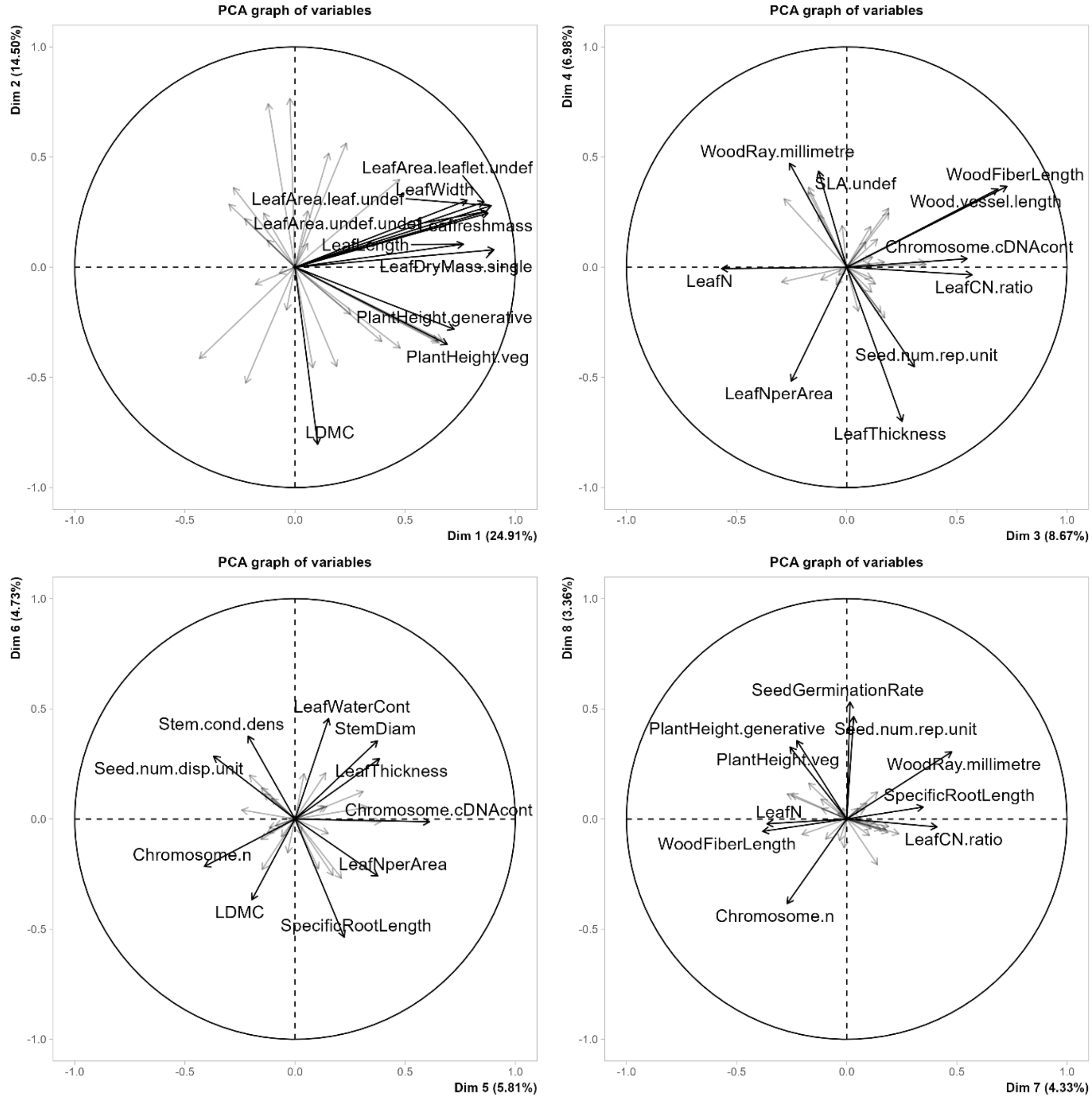

Supplementary Fig. 12. The first eight principal components in the joint relationships among the 46 traits of the gap-filled TRY plant trait database (Kattge et al. 2020), calculated for the subset of 3,834 taxa that occurred in our vegetation-plot time series data. Trait names and descriptions are noted in Extended Data Table 1. For each pair of principal components, the 10 traits with the strongest relationships are shown in black; the less weakly related traits are only indicated with grey arrows.

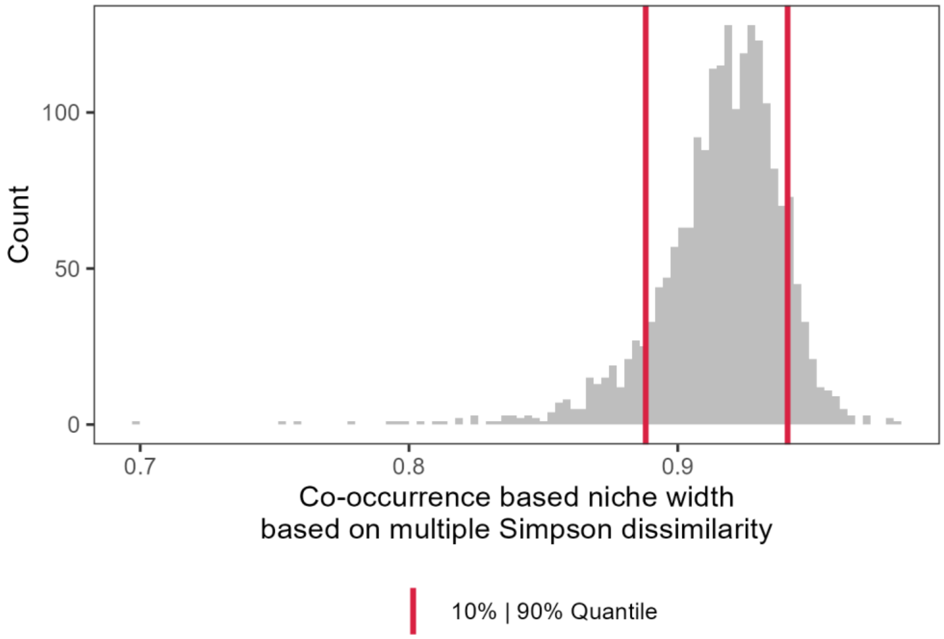

Supplementary Fig. 13. Distribution of relative niche width estimates for 1,995 species with ≥ 50 occurrences in vegetation-plot observations, together with limits of 10% and 90% quantiles that were used to separate habitat-specialists (to the left) and habitat-generalists (to the right).
