# Supplementary material for: Habitat-specific trends in taxonomic, functional, and phylogenetic diversity in European plant communities over a century
Source: Nat Commun. 2026 May 8;17:4208. doi: 10.1038/s41467-026-72112-5 (PMC13156317; doi:10.1038/s41467-026-72112-5)
Supplement: Supplementary file 2 — Description of Additional Supplementary Information [file 41467_2026_72112_MOESM2_ESM.pdf]

Supplementary Data 1. EUNIS habitat names and samples sizes (number of vegetation-plot observations). Observations that could be assigned to multiple EUNIS habitats were assigned at the most narrowly matching level (i.e., to level 2 or 1). Sample sizes for level 1 and 2 habitat types only include observations that could not be classified into more narrowly defined habitat types. For distributions of time series see Supplementary Fig. 1.

Supplementary Data 2. Species phylogenetic relationships. Phylogeny was extracted from the sPlot database, version 3.0 [20,100] — a super tree based on the Open Tree of Life [40] with 589 additional species on 634 nodes that sum up to 7,857 species. Taxa that were not resolved by the phylogenies of origin were bound to the most recent common ancestor if the genus included more than one species, or to half of the terminal level of a sister species if only one species was available in the focal genus.

Supplementary Data 3. Expert-based classification of habitat change trajectories.

Supplementary Data 4. Average annual percentage changes in diversity indices of local plant communities before versus after the year 2000.

Supplementary Data 5. Statistics for the analyses of explained variation in biodiversity trends via EUNIS level 3 habitat, habitat-change trajectory, and last observation year. Results are illustrated in Fig. 4.

Supplementary Data 6. Statistics for the specific trends in biodiversity indices per EUNIS level 1 habitat and habitat-change trajectory. Results are illustrated in Supplementary Figs. 7–8.

Supplementary Data 7. Statistics for the analyses of habitat-specific gamma diversity. Results are illustrated in Fig. 6 and Supplementary Fig. 9.

Supplementary Data 8. Statistics for the analyses of influence of plot size on habitat-specific trends in plant diversity. Results are illustrated in Supplementary Fig. 10.

Supplementary Data 9. Individual vegetation databases used for this study. Raw data can be requested at the European Vegetation Archive (euroveg.org).
