# Supplementary figures and images for: Habitat-specific trends in taxonomic, functional, and phylogenetic diversity in European plant communities over a century
Source: Nat Commun. 2026 May 8;17:4208. doi: 10.1038/s41467-026-72112-5 (PMC13156317; doi:10.1038/s41467-026-72112-5)

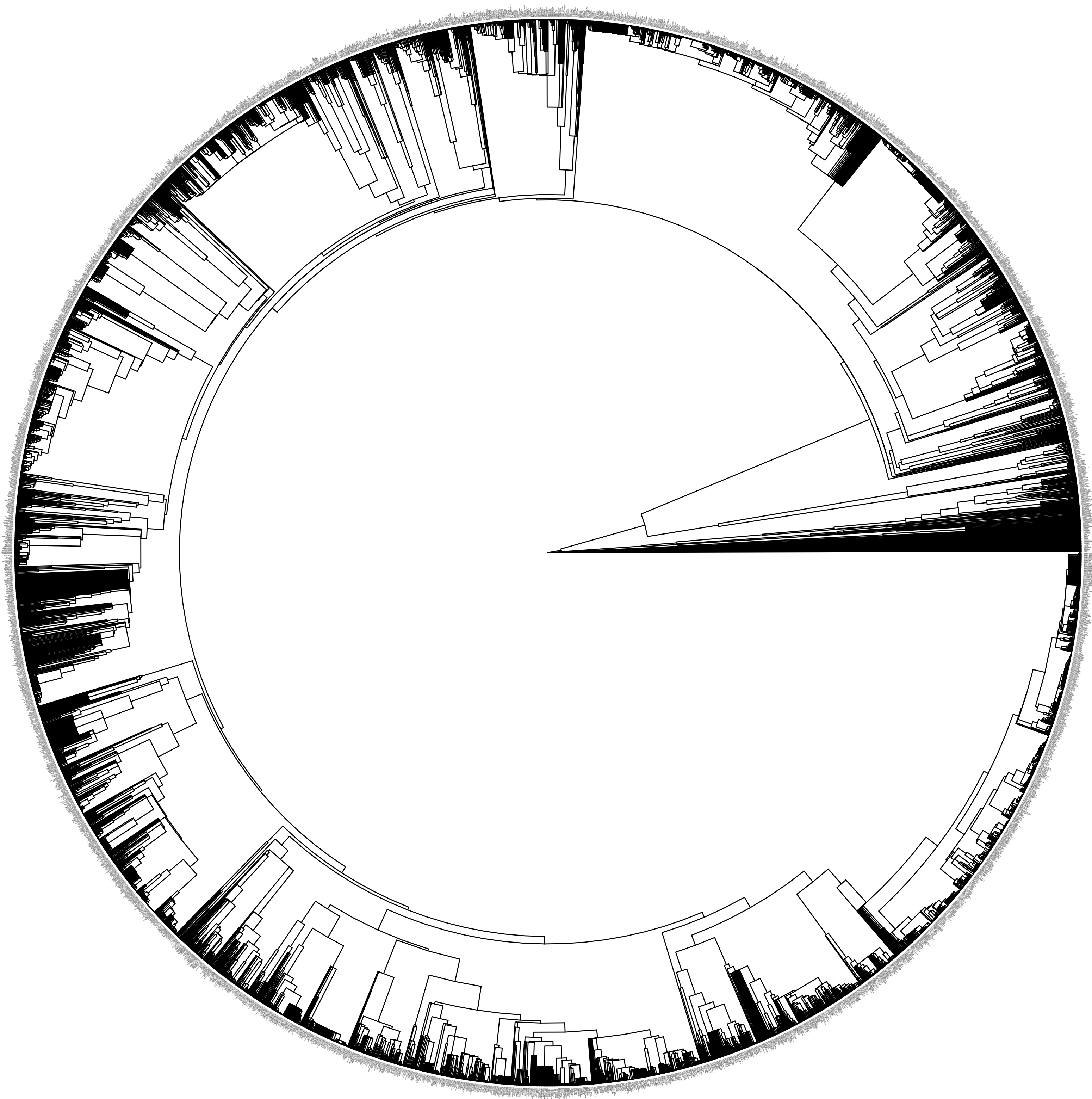

Supplement: Supplementary file 4 — Supplementary Data 2 [file 41467_2026_72112_MOESM4_ESM.pdf]
