# Supplementary material for: Habitat-specific trends in taxonomic, functional, and phylogenetic diversity in European plant communities over a century
Source: Nat Commun. 2026 May 8;17:4208. doi: 10.1038/s41467-026-72112-5 (PMC13156317; doi:10.1038/s41467-026-72112-5)
Supplement: Supplementary file 12 — Reporting Summary [file 41467_2026_72112_MOESM12_ESM.pdf]

nature portfolio

Corresponding author(s): Stephan Kambach

Last updated by author(s): Mar 27, 2026

Reporting Summary

Nature Portfolio wishes to improve the reproducibility of the work that we publish. This form provides structure for consistency and transparency in reporting. For further information on Nature Portfolio policies, see our Editorial Policies and the Editorial Policy Checklist.

Statistics

For all statistical analyses, confirm that the following items are present in the figure legend, table legend, main text, or Methods section.

n/a	Confirmed	
☐	☒	The exact sample size (*n*) for each experimental group/condition, given as a discrete number and unit of measurement
☐	☒	A statement on whether measurements were taken from distinct samples or whether the same sample was measured repeatedly
☐	☒	The statistical test(s) used AND whether they are one- or two-sided<br>*Only common tests should be described solely by name; describe more complex techniques in the Methods section.*
☐	☒	A description of all covariates tested
☒	☐	A description of any assumptions or corrections, such as tests of normality and adjustment for multiple comparisons
☐	☒	A full description of the statistical parameters including central tendency (e.g. means) or other basic estimates (e.g. regression coefficient) AND variation (e.g. standard deviation) or associated estimates of uncertainty (e.g. confidence intervals)
☐	☒	For null hypothesis testing, the test statistic (e.g. *F*, *t*, *r*) with confidence intervals, effect sizes, degrees of freedom and *P* value noted<br>*Give P values as exact values whenever suitable.*
☒	☐	For Bayesian analysis, information on the choice of priors and Markov chain Monte Carlo settings
☐	☒	For hierarchical and complex designs, identification of the appropriate level for tests and full reporting of outcomes
☒	☐	Estimates of effect sizes (e.g. Cohen's *d*, Pearson's *r*), indicating how they were calculated

Our web collection on statistics for biologists contains articles on many of the points above.

Software and code

Policy information about availability of computer code

Data collection	Vegetation survey data was provided by the European Vegetation Archive (euroveg.org), where raw data was processed and harmonized with with Turboveg 2 (www.synbiosys.alterra.nl/turboveg) and Juice 7.0 (www.sci.muni.cz/botany/juice). Trait data was exported from the TRY Plant Trait Database, version 6.0 (www.try-db.org) and processed by the sPlot – Global Vegetation Databasen 4.0 (www.idiv.de/research/projects/splot). For this study we harmonized taxa names with the World Flora Online Database 2023.12 (www.worldfloraonline.org) and further processed all data with with R 4.0 running in RStudio 2025.5 (posit.co/download/rstudio-desktop).
Data analysis	All analyses were conducted in R (version 4.0), using the packages dominanceanalysis (2.1.1), picante (1.8.2), FactoMineR (2.12), data.tree (1.2.0), ggplot2 (3.5.2), ggalluvial (012.5), ggh4x (0.3.1), networkD3 (0.4.1), rnaturalearth (1.1.0), rnaturalearthdata (1.0.0), weights (1.1.2), and WorldFlora (1.14-5).<br>The R-code generated for data manipulation, analysis, and visualisation is openly available at:<br>github.com/StephanKambach/Local_trends_in_plant_diversity

For manuscripts utilizing custom algorithms or software that are central to the research but not yet described in published literature, software must be made available to editors and reviewers. We strongly encourage code deposition in a community repository (e.g. GitHub). See the Nature Portfolio guidelines for submitting code & software for further information.

Data

Policy information about availability of data

All manuscripts must include a data availability statement. This statement should provide the following information, where applicable:

- Accession codes, unique identifiers, or web links for publicly available datasets
- A description of any restrictions on data availability
- For clinical datasets or third party data, please ensure that the statement adheres to our policy

The data generated in this study, i.e. time series-specific linear trends in all biodiversity indices, expressed in annual percentage changes and in absolute units are openly available at the data repository of the German Centre for Integrated Biodiversity Research (iDiv) Halle-Jena-Leipzig (idata.idiv.de/ddm/Data/ShowData/3611, DOI 10.25829/idiv.3611-rgwa69). Raw vegetation data cannot be made openly available as they belong to the owners and custodians of each vegetation database — but can be requested at the European Vegetation Archive 123. Individual vegetation databases for this study are listed in Supplementary Data 9. Plant trait data can be downloaded at the website of the TRY plant trait database (www.try-db.org). Phylogenetic data can be downloaded at the website of the Open Tree of Life (tree.opentreeoflife.org). Classification into native and non-native species is openly available at the FloraVeg.EU database (floraveg.eu). Red List data was downloaded from github.com/istaude/european-redlist-synthesis. A harmonized version can be requested from Laura Méndez (laura.mendez@ufz.de).

Research involving human participants, their data, or biological material

Policy information about studies with human participants or human data. See also policy information about sex, gender (identity/presentation), and sexual orientation and race, ethnicity and racism.

Reporting on sex and gender	Not applicable.
Reporting on race, ethnicity, or other socially relevant groupings	*Please specify the socially constructed or socially relevant categorization variable(s) used in your manuscript and explain why they were used. Please note that such variables should not be used as proxies for other socially constructed/relevant variables (for example, race or ethnicity should not be used as a proxy for socioeconomic status).* *Provide clear definitions of the relevant terms used, how they were provided (by the participants/respondents, the researchers, or third parties), and the method(s) used to classify people into the different categories (e.g. self-report, census or administrative data, social media data, etc.)* *Please provide details about how you controlled for confounding variables in your analyses.*
Population characteristics	Not applicable.
Recruitment	Not applicable.
Ethics oversight	Not applicable.

Note that full information on the approval of the study protocol must also be provided in the manuscript.

Field-specific reporting

Please select the one below that is the best fit for your research. If you are not sure, read the appropriate sections before making your selection.

☐ Life sciences ☐ Behavioural & social sciences ☒ Ecological, evolutionary & environmental sciences

For a reference copy of the document with all sections, see nature.com/documents/nr-reporting-summary-flat.pdf

Ecological, evolutionary & environmental sciences study design

All studies must disclose on these points even when the disclosure is negative.

Study description	For this study, we analysed long-term trend in local European plant communities using 199,282 vegetation surveys from 57,390 time series of repeated vegetation surveys of permanent or quasi-permanent plots. For each vegetation survey, we calculated the summed vegetation cover, two indices of taxonomic diversity, three indices of functional diversity, three indices of phylogenetic diversity, and two indices of threatened species. All vegetation surveys were assigned to EUNIS habitat types (based on floristic composition and location) and every time series was assigned to a stable, successional, or disturbance trajectory (based on changes in EUNIS habitat types). Using weighted linear and mixed-effects models, we then calculated the linear annual trends in these diversity indices and the summed number of taxa observed among different decades - all across and within EUNIS level 1 habtiat types.
Research sample	Raw vegetation data consisted of 429,917 vegetation surveys from 119,964 permanent and semi-permanent vegetation plots provided by the European Vegetation Archive on April 4th, 2024 (project #200).
Sampling strategy	We aimed to include all available vegetation time series with repeated plot observations that noted species abundance/cover values. We further aimed at using a comprehensive set of gap-filled traits, a global phylogeny, and harmonized European Red List data to calculate diverstity indices for as many vegetation surveys as possible (with at least 80% of area covered by species with the respective information).
Data collection	Vegetation data were collected by trained field Botanists that noted presence/absence and cover/abundance of all vascular plant

	taxa in the target plot locations (recorded with pen and paper, transfered to spreadsheet, and then sent to the European Vegetation Archive).
Timing and spatial scale	Vegetation survey data included observations from 1911 - 2024 and distributed between -9.041862 and 44.51 Longitude and between 35.26861 and 71.04241 Latitude.
Data exclusions	We excluded plots that were experimentally manipulated for research purposes but kept all plots that were regularly mown or managed following traditional or regular management practices. We excluded all surveys that consisted only of species presence/absence data without species cover information because this data did not permit the assignment of EUNIS habitat types or the calculation of most indices of plant diversity. We further excluded all time series in which the area of the vegetation plot changed by a factor of more than two between subsequent surveys. The filtered dataset included 199,282 vegetation surveys belonging to 57,390 different time series.
Reproducibility	We provide an analysis of observational data. Tests of reproducibility thus will only be possible when novel data enters the European Vegetation Archive.
Randomization	Not applicable.
Blinding	Not applicable.

Did the study involve field work? ☒ Yes ☐ No

Field work, collection and transport

Field conditions	Not applicable as these factors were not provided by the initial data collectors.
Location	European terrestrial habitats.
Access & import/export	Data access was requested and provided in accordance with the regulations of the European Vegetation Archive and the ReSurveyEUROPE initiative (euroveg.org/eva-database/obtaining-data).
Disturbance	Whenever disturbances were noted by the original data collectors, these vegetation surveys were omitted from the analysis.

Reporting for specific materials, systems and methods

We require information from authors about some types of materials, experimental systems and methods used in many studies. Here, indicate whether each material, system or method listed is relevant to your study. If you are not sure if a list item applies to your research, read the appropriate section before selecting a response.

Materials & experimental systems

n/a	Involved in the study	
☒	☐	Antibodies
☒	☐	Eukaryotic cell lines
☒	☐	Palaeontology and archaeology
☒	☐	Animals and other organisms
☒	☐	Clinical data
☒	☐	Dual use research of concern
☒	☐	Plants

Methods

n/a	Involved in the study	
☒	☐	ChIP-seq
☒	☐	Flow cytometry
☒	☐	MRI-based neuroimaging

Plants

Seed stocks	Not applicable.
Novel plant genotypes	Not applicable.
Authentication	Not applicable.
